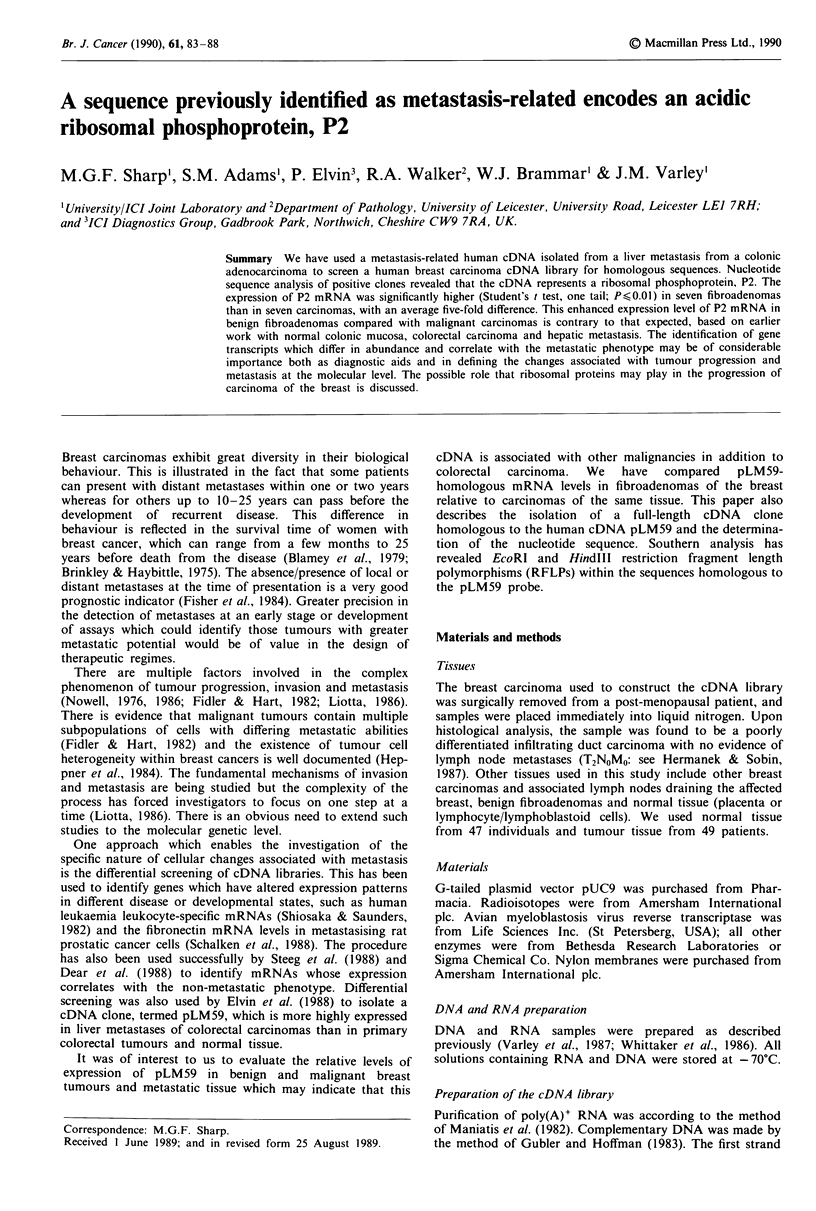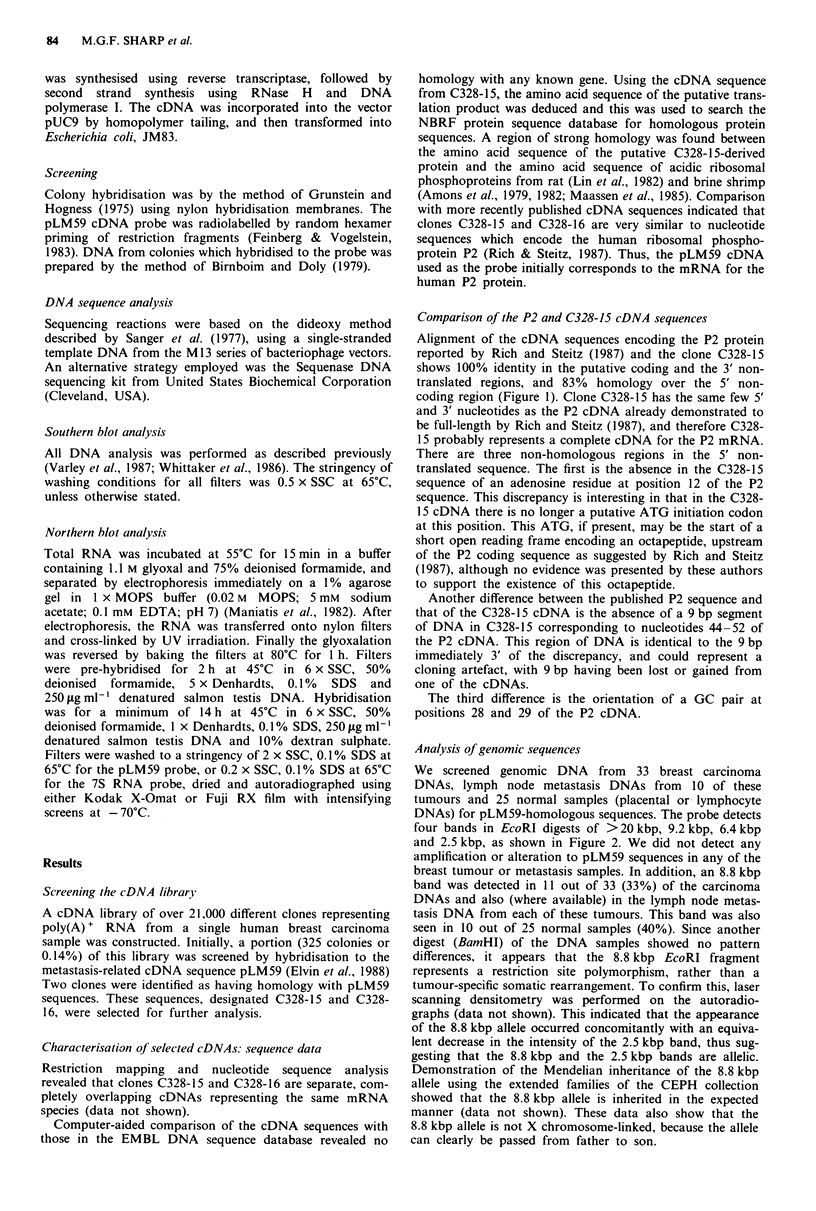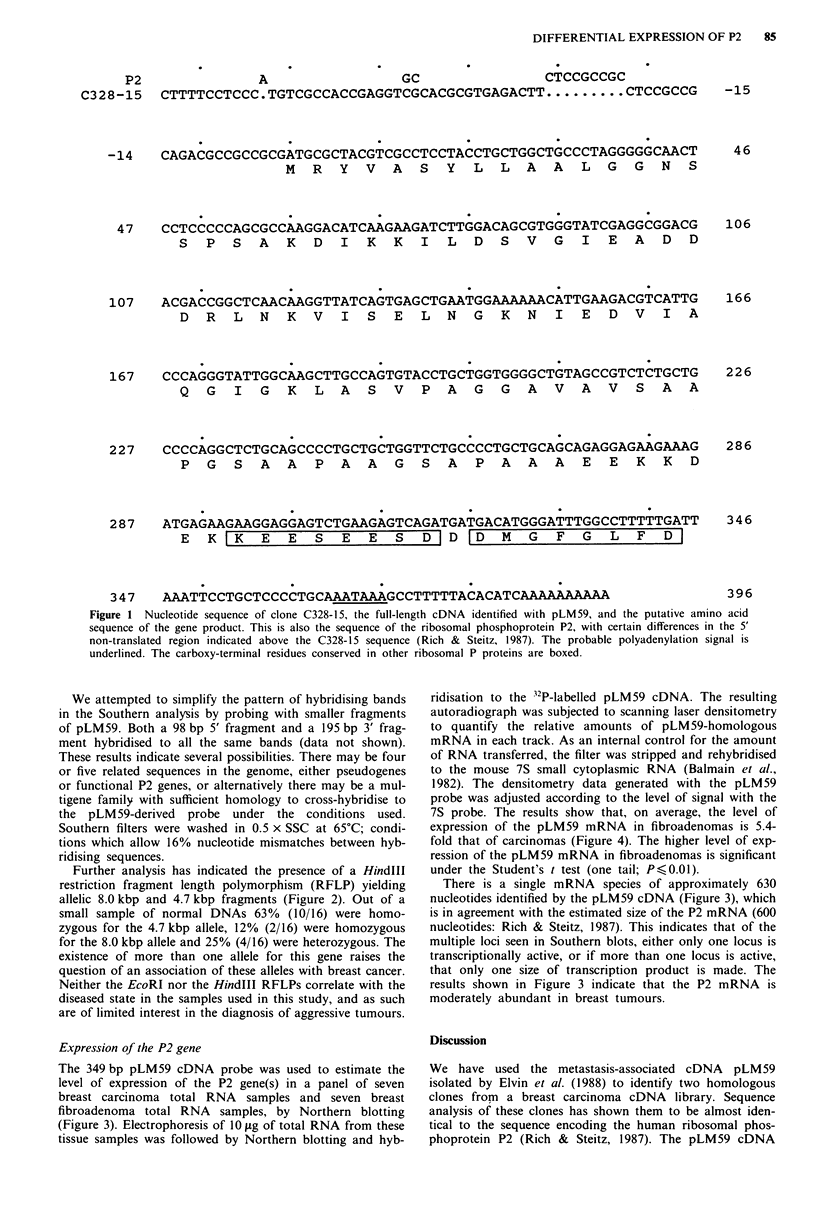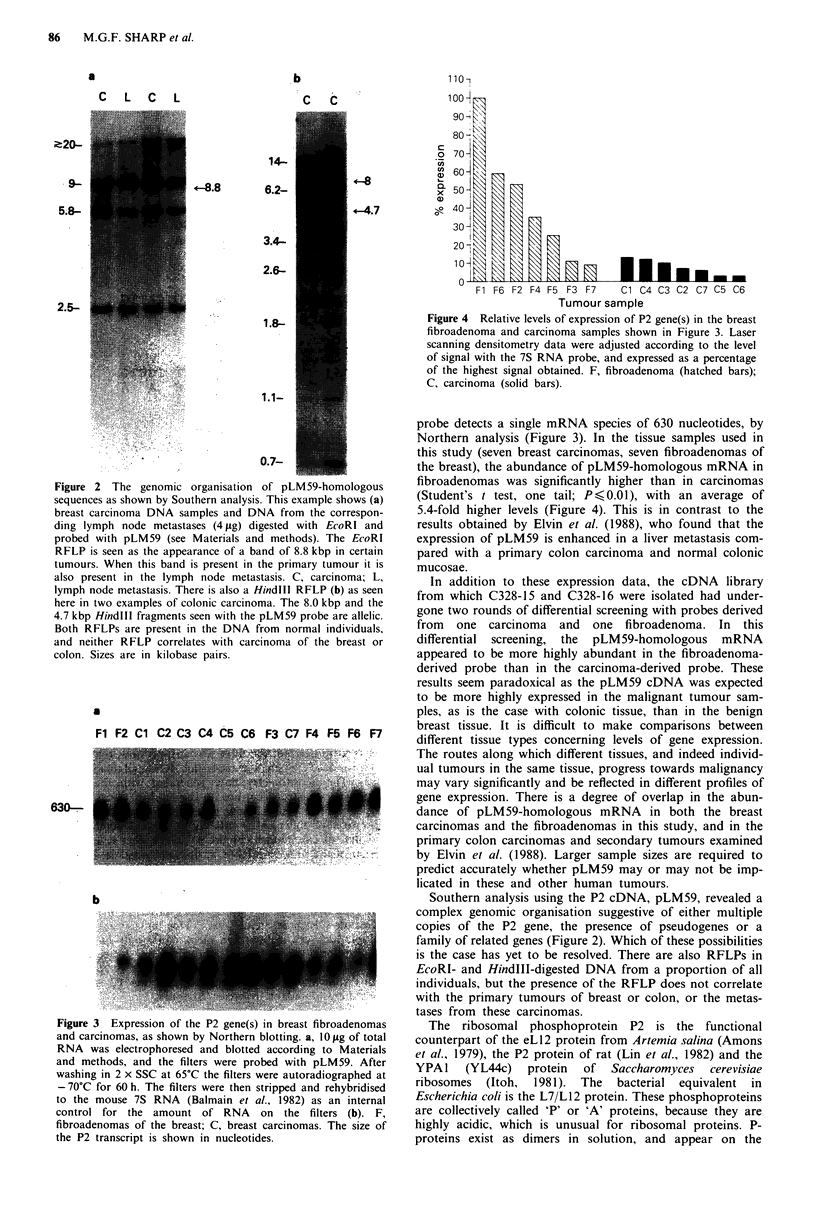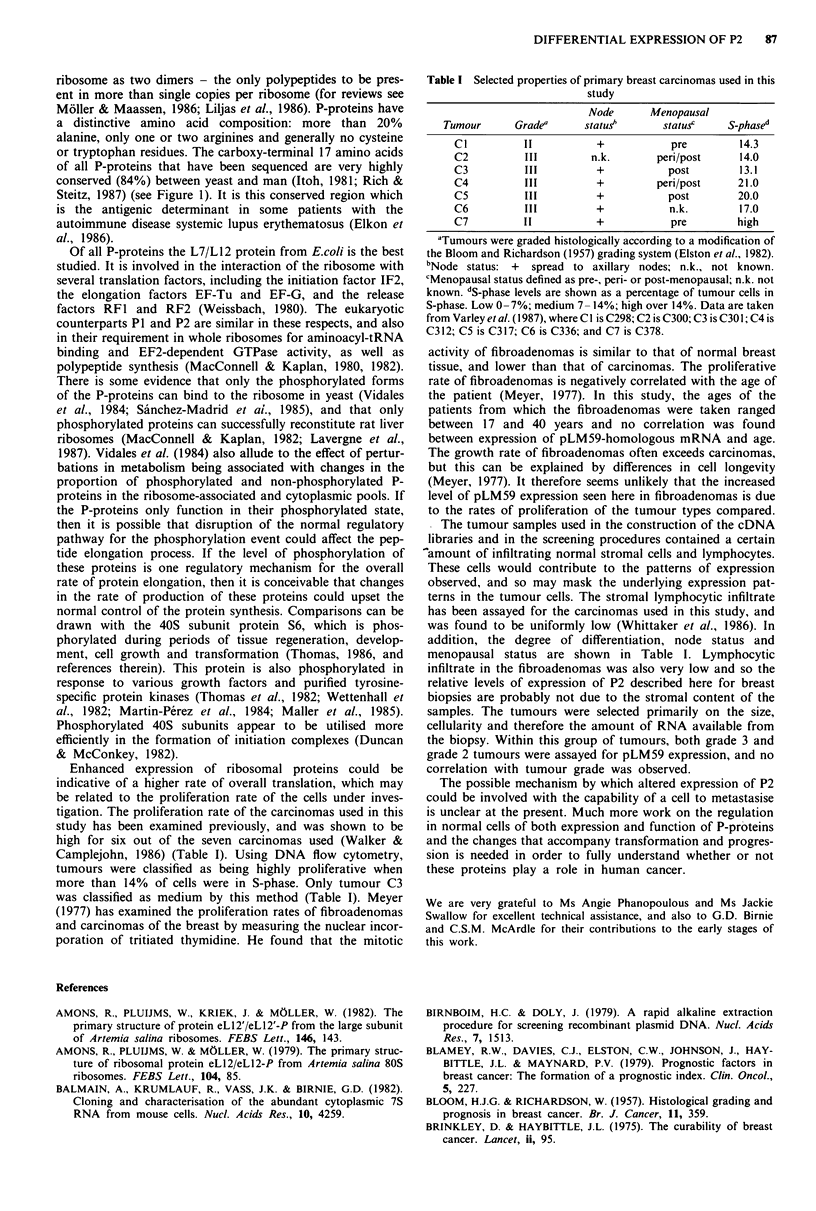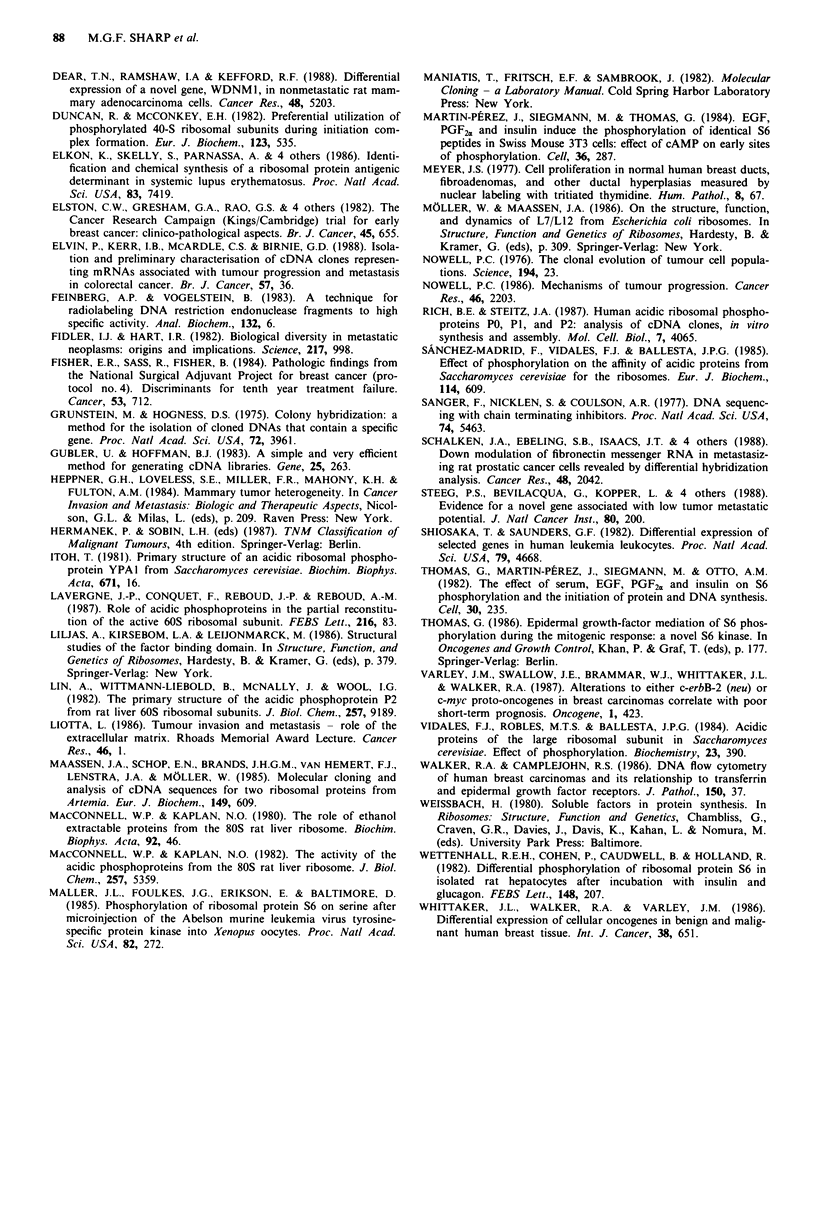# A sequence previously identified as metastasis-related encodes an acidic ribosomal phosphoprotein, P2.

**DOI:** 10.1038/bjc.1990.19

**Published:** 1990-01

**Authors:** M. G. Sharp, S. M. Adams, P. Elvin, R. A. Walker, W. J. Brammar, J. M. Varley

**Affiliations:** University/ICI Joint Laboratory, Leicester, UK.

## Abstract

**Images:**


Br. J. Cancer (1990), 61, 83–88
© Macmillan Press Ltd., 1990

### A sequence previously identified as metastasis-related encodes an acidic ribosomal phosphoprotein, P2

M.G.F. Sharp[1], S.M. Adams[1], P. Elvin[3], R.A. Walker[2], W.J. Brammar[1] & J.M. Varley[1]

[1]*University/ICI Joint Laboratory and* [2]*Department of Pathology, University of Leicester, University Road, Leicester LE1 7RH; and* [3]*ICI Diagnostics Group, Gadbrook Park, Northwich, Cheshire CW9 7RA, UK.*

**Summary** We have used a metastasis-related human cDNA isolated from a liver metastasis from a colonic adenocarcinoma to screen a human breast carcinoma cDNA library for homologous sequences. Nucleotide sequence analysis of positive clones revealed that the cDNA represents a ribosomal phosphoprotein, P2. The expression of P2 mRNA was significantly higher (Student's $t$ test, one tail; $P \leqslant 0.01$) in seven fibroadenomas than in seven carcinomas, with an average five-fold difference. This enhanced expression level of P2 mRNA in benign fibroadenomas compared with malignant carcinomas is contrary to that expected, based on earlier work with normal colonic mucosa, colorectal carcinoma and hepatic metastasis. The identification of gene transcripts which differ in abundance and correlate with the metastatic phenotype may be of considerable importance both as diagnostic aids and in defining the changes associated with tumour progression and metastasis at the molecular level. The possible role that ribosomal proteins may play in the progression of carcinoma of the breast is discussed.

Breast carcinomas exhibit great diversity in their biological behaviour. This is illustrated in the fact that some patients can present with distant metastases within one or two years whereas for others up to 10–25 years can pass before the development of recurrent disease. This difference in behaviour is reflected in the survival time of women with breast cancer, which can range from a few months to 25 years before death from the disease (Blamey *et al.*, 1979; Brinkley & Haybittle, 1975). The absence/presence of local or distant metastases at the time of presentation is a very good prognostic indicator (Fisher *et al.*, 1984). Greater precision in the detection of metastases at an early stage or development of assays which could identify those tumours with greater metastatic potential would be of value in the design of therapeutic regimes.

There are multiple factors involved in the complex phenomenon of tumour progression, invasion and metastasis (Nowell, 1976, 1986; Fidler & Hart, 1982; Liotta, 1986). There is evidence that malignant tumours contain multiple subpopulations of cells with differing metastatic abilities (Fidler & Hart, 1982) and the existence of tumour cell heterogeneity within breast cancers is well documented (Heppner *et al.*, 1984). The fundamental mechanisms of invasion and metastasis are being studied but the complexity of the process has forced investigators to focus on one step at a time (Liotta, 1986). There is an obvious need to extend such studies to the molecular genetic level.

One approach which enables the investigation of the specific nature of cellular changes associated with metastasis is the differential screening of cDNA libraries. This has been used to identify genes which have altered expression patterns in different disease or developmental states, such as human leukaemia leukocyte-specific mRNAs (Shiosaka & Saunders, 1982) and the fibronectin mRNA levels in metastasising rat prostatic cancer cells (Schalken *et al.*, 1988). The procedure has also been used successfully by Steeg *et al.* (1988) and Dear *et al.* (1988) to identify mRNAs whose expression correlates with the non-metastatic phenotype. Differential screening was also used by Elvin *et al.* (1988) to isolate a cDNA clone, termed pLM59, which is more highly expressed in liver metastases of colorectal carcinomas than in primary colorectal tumours and normal tissue.

It was of interest to us to evaluate the relative levels of expression of pLM59 in benign and malignant breast tumours and metastatic tissue which may indicate that this cDNA is associated with other malignancies in addition to colorectal carcinoma. We have compared pLM59-homologous mRNA levels in fibroadenomas of the breast relative to carcinomas of the same tissue. This paper also describes the isolation of a full-length cDNA clone homologous to the human cDNA pLM59 and the determination of the nucleotide sequence. Southern analysis has revealed *Eco*RI and *Hin*dIII restriction fragment length polymorphisms (RFLPs) within the sequences homologous to the pLM59 probe.

Correspondence: M.G.F. Sharp.
Received 1 June 1989; and in revised form 25 August 1989.

### Materials and methods

### *Tissues*

The breast carcinoma used to construct the cDNA library was surgically removed from a post-menopausal patient, and samples were placed immediately into liquid nitrogen. Upon histological analysis, the sample was found to be a poorly differentiated infiltrating duct carcinoma with no evidence of lymph node metastases ($T_2N_0M_0$: see Hermanek & Sobin, 1987). Other tissues used in this study include other breast carcinomas and associated lymph nodes draining the affected breast, benign fibroadenomas and normal tissue (placenta or lymphocyte/lymphoblastoid cells). We used normal tissue from 47 individuals and tumour tissue from 49 patients.

### *Materials*

G-tailed plasmid vector pUC9 was purchased from Pharmacia. Radioisotopes were from Amersham International plc. Avian myeloblastosis virus reverse transcriptase was from Life Sciences Inc. (St Petersberg, USA); all other enzymes were from Bethesda Research Laboratories or Sigma Chemical Co. Nylon membranes were purchased from Amersham International plc.

### *DNA and RNA preparation*

DNA and RNA samples were prepared as described previously (Varley *et al.*, 1987; Whittaker *et al.*, 1986). All solutions containing RNA and DNA were stored at −70°C.

### *Preparation of the cDNA library*

Purification of poly(A)$^+$ RNA was according to the method of Maniatis *et al.* (1982). Complementary DNA was made by the method of Gubler and Hoffman (1983). The first strand was synthesised using reverse transcriptase, followed by second strand synthesis using RNase H and DNA polymerase I. The cDNA was incorporated into the vector pUC9 by homopolymer tailing, and then transformed into *Escherichia coli*, JM83.

*Screening*

Colony hybridisation was by the method of Grunstein and Hogness (1975) using nylon hybridisation membranes. The pLM59 cDNA probe was radiolabelled by random hexamer priming of restriction fragments (Feinberg & Vogelstein, 1983). DNA from colonies which hybridised to the probe was prepared by the method of Birnboim and Doly (1979).

*DNA sequence analysis*

Sequencing reactions were based on the dideoxy method described by Sanger *et al.* (1977), using a single-stranded template DNA from the M13 series of bacteriophage vectors. An alternative strategy employed was the Sequenase DNA sequencing kit from United States Biochemical Corporation (Cleveland, USA).

*Southern blot analysis*

All DNA analysis was performed as described previously (Varley *et al.*, 1987; Whittaker *et al.*, 1986). The stringency of washing conditions for all filters was 0.5 × SSC at 65°C, unless otherwise stated.

*Northern blot analysis*

Total RNA was incubated at 55°C for 15 min in a buffer containing 1.1 M glyoxal and 75% deionised formamide, and separated by electrophoresis immediately on a 1% agarose gel in 1 × MOPS buffer (0.02 M MOPS; 5 mM sodium acetate; 0.1 mM EDTA; pH 7) (Maniatis *et al.*, 1982). After electrophoresis, the RNA was transferred onto nylon filters and cross-linked by UV irradiation. Finally the glyoxalation was reversed by baking the filters at 80°C for 1 h. Filters were pre-hybridised for 2 h at 45°C in 6 × SSC, 50% deionised formamide, 5 × Denhardts, 0.1% SDS and 250 $\mu g\ ml^{-1}$ denatured salmon testis DNA. Hybridisation was for a minimum of 14 h at 45°C in 6 × SSC, 50% deionised formamide, 1 × Denhardts, 0.1% SDS, 250 $\mu g\ ml^{-1}$ denatured salmon testis DNA and 10% dextran sulphate. Filters were washed to a stringency of 2 × SSC, 0.1% SDS at 65°C for the pLM59 probe, or 0.2 × SSC, 0.1% SDS at 65°C for the 7S RNA probe, dried and autoradiographed using either Kodak X-Omat or Fuji RX film with intensifying screens at −70°C.

### Results

*Screening the cDNA library*

A cDNA library of over 21,000 different clones representing poly(A)$^+$ RNA from a single human breast carcinoma sample was constructed. Initially, a portion (325 colonies or 0.14%) of this library was screened by hybridisation to the metastasis-related cDNA sequence pLM59 (Elvin *et al.*, 1988) Two clones were identified as having homology with pLM59 sequences. These sequences, designated C328-15 and C328-16, were selected for further analysis.

*Characterisation of selected cDNAs: sequence data*

Restriction mapping and nucleotide sequence analysis revealed that clones C328-15 and C328-16 are separate, completely overlapping cDNAs representing the same mRNA species (data not shown).

Computer-aided comparison of the cDNA sequences with those in the EMBL DNA sequence database revealed no homology with any known gene. Using the cDNA sequence from C328-15, the amino acid sequence of the putative translation product was deduced and this was used to search the NBRF protein sequence database for homologous protein sequences. A region of strong homology was found between the amino acid sequence of the putative C328-15-derived protein and the amino acid sequence of acidic ribosomal phosphoproteins from rat (Lin *et al.*, 1982) and brine shrimp (Amons *et al.*, 1979, 1982; Maassen *et al.*, 1985). Comparison with more recently published cDNA sequences indicated that clones C328-15 and C328-16 are very similar to nucleotide sequences which encode the human ribosomal phosphoprotein P2 (Rich & Steitz, 1987). Thus, the pLM59 cDNA used as the probe initially corresponds to the mRNA for the human P2 protein.

*Comparison of the P2 and C328-15 cDNA sequences*

Alignment of the cDNA sequences encoding the P2 protein reported by Rich and Steitz (1987) and the clone C328-15 shows 100% identity in the putative coding and the 3′ non-translated regions, and 83% homology over the 5′ non-coding region (Figure 1). Clone C328-15 has the same few 5′ and 3′ nucleotides as the P2 cDNA already demonstrated to be full-length by Rich and Steitz (1987), and therefore C328-15 probably represents a complete cDNA for the P2 mRNA. There are three non-homologous regions in the 5′ non-translated sequence. The first is the absence in the C328-15 sequence of an adenosine residue at position 12 of the P2 sequence. This discrepancy is interesting in that in the C328-15 cDNA there is no longer a putative ATG initiation codon at this position. This ATG, if present, may be the start of a short open reading frame encoding an octapeptide, upstream of the P2 coding sequence as suggested by Rich and Steitz (1987), although no evidence was presented by these authors to support the existence of this octapeptide.

Another difference between the published P2 sequence and that of the C328-15 cDNA is the absence of a 9 bp segment of DNA in C328-15 corresponding to nucleotides 44–52 of the P2 cDNA. This region of DNA is identical to the 9 bp immediately 3′ of the discrepancy, and could represent a cloning artefact, with 9 bp having been lost or gained from one of the cDNAs.

The third difference is the orientation of a GC pair at positions 28 and 29 of the P2 cDNA.

*Analysis of genomic sequences*

We screened genomic DNA from 33 breast carcinoma DNAs, lymph node metastasis DNAs from 10 of these tumours and 25 normal samples (placental or lymphocyte DNAs) for pLM59-homologous sequences. The probe detects four bands in *Eco*RI digests of >20 kbp, 9.2 kbp, 6.4 kbp and 2.5 kbp, as shown in Figure 2. We did not detect any amplification or alteration to pLM59 sequences in any of the breast tumour or metastasis samples. In addition, an 8.8 kbp band was detected in 11 out of 33 (33%) of the carcinoma DNAs and also (where available) in the lymph node metastasis DNA from each of these tumours. This band was also seen in 10 out of 25 normal samples (40%). Since another digest (*Bam*HI) of the DNA samples showed no pattern differences, it appears that the 8.8 kbp *Eco*RI fragment represents a restriction site polymorphism, rather than a tumour-specific somatic rearrangement. To confirm this, laser scanning densitometry was performed on the autoradiographs (data not shown). This indicated that the appearance of the 8.8 kbp allele occurred concomitantly with an equivalent decrease in the intensity of the 2.5 kbp band, thus suggesting that the 8.8 kbp and the 2.5 kbp bands are allelic. Demonstration of the Mendelian inheritance of the 8.8 kbp allele using the extended families of the CEPH collection showed that the 8.8 kbp allele is inherited in the expected manner (data not shown). These data also show that the 8.8 kbp allele is not X chromosome-linked, because the allele can clearly be passed from father to son.

```
             .         .         .         .         .         .
P2                  A               GC              CTCCGCCGC
C328-15  CTTTTCCTCCC.TGTCGCCACCGAGGTCGCACGCGTGAGACTT.........CTCCGCCG   -15

             .         .         .         .         .         .
-14      CAGACGCCGCCGCGATGCGCTACGTCGCCTCCTACCTGCTGGCTGCCCTAGGGGGCAACT   46
                        M  R  Y  V  A  S  Y  L  L  A  A  L  G  G  N  S

             .         .         .         .         .         .
47       CCTCCCCCAGCGCCAAGGACATCAAGAAGATCTTGGACAGCGTGGGTATCGAGGCGGACG   106
           S  P  S  A  K  D  I  K  K  I  L  D  S  V  G  I  E  A  D  D

             .         .         .         .         .         .
107      ACGACCGGCTCAACAAGGTTATCAGTGAGCTGAATGGAAAAAACATTGAAGACGTCATTG   166
           D  R  L  N  K  V  I  S  E  L  N  G  K  N  I  E  D  V  I  A

             .         .         .         .         .         .
167      CCCAGGGTATTGGCAAGCTTGCCAGTGTACCTGCTGGTGGGGCTGTAGCCGTCTCTGCTG   226
           Q  G  I  G  K  L  A  S  V  P  A  G  G  A  V  A  V  S  A  A

             .         .         .         .         .         .
227      CCCCAGGCTCTGCAGCCCCTGCTGCTGGTTCTGCCCCTGCTGCAGCAGAGGAGAAGAAAG   286
           P  G  S  A  A  P  A  A  G  S  A  P  A  A  A  E  E  K  K  D

             .         .         .         .         .         .

287      ATGAGAAGAAGGAGGAGTCTGAAGAGTCAGATGATGACATGGGATTTGGCCTTTTTGATT   346

           E  K [K  E  E  S  E  E  S  D] D [D  M  G  F  G  L  F  D]

             .         .         .         .         .
347      AAATTCCTGCTCCCCTGCAAATAAAGCCTTTTTACACATCAAAAAAAAAA             396
```

**Figure 1** Nucleotide sequence of clone C328-15, the full-length cDNA identified with pLM59, and the putative amino acid sequence of the gene product. This is also the sequence of the ribosomal phosphoprotein P2, with certain differences in the 5′ non-translated region indicated above the C328-15 sequence (Rich & Steitz, 1987). The probable polyadenylation signal is underlined. The carboxy-terminal residues conserved in other ribosomal P proteins are boxed.

We attempted to simplify the pattern of hybridising bands in the Southern analysis by probing with smaller fragments of pLM59. Both a 98 bp 5′ fragment and a 195 bp 3′ fragment hybridised to all the same bands (data not shown). These results indicate several possibilities. There may be four or five related sequences in the genome, either pseudogenes or functional P2 genes, or alternatively there may be a multigene family with sufficient homology to cross-hybridise to the pLM59-derived probe under the conditions used. Southern filters were washed in 0.5 × SSC at 65°C; conditions which allow 16% nucleotide mismatches between hybridising sequences.

Further analysis has indicated the presence of a *Hin*dIII restriction fragment length polymorphism (RFLP) yielding allelic 8.0 kbp and 4.7 kbp fragments (Figure 2). Out of a small sample of normal DNAs 63% (10/16) were homozygous for the 4.7 kbp allele, 12% (2/16) were homozygous for the 8.0 kbp allele and 25% (4/16) were heterozygous. The existence of more than one allele for this gene raises the question of an association of these alleles with breast cancer. Neither the *Eco*RI nor the *Hin*dIII RFLPs correlate with the diseased state in the samples used in this study, and as such are of limited interest in the diagnosis of aggressive tumours.

*Expression of the P2 gene*

The 349 bp pLM59 cDNA probe was used to estimate the level of expression of the P2 gene(s) in a panel of seven breast carcinoma total RNA samples and seven breast fibroadenoma total RNA samples, by Northern blotting (Figure 3). Electrophoresis of 10 μg of total RNA from these tissue samples was followed by Northern blotting and hybridisation to the $^{32}$P-labelled pLM59 cDNA. The resulting autoradiograph was subjected to scanning laser densitometry to quantify the relative amounts of pLM59-homologous mRNA in each track. As an internal control for the amount of RNA transferred, the filter was stripped and rehybridised to the mouse 7S small cytoplasmic RNA (Balmain *et al.*, 1982). The densitometry data generated with the pLM59 probe was adjusted according to the level of signal with the 7S probe. The results show that, on average, the level of expression of the pLM59 mRNA in fibroadenomas is 5.4-fold that of carcinomas (Figure 4). The higher level of expression of the pLM59 mRNA in fibroadenomas is significant under the Student's *t* test (one tail; $P \leqslant 0.01$).

There is a single mRNA species of approximately 630 nucleotides identified by the pLM59 cDNA (Figure 3), which is in agreement with the estimated size of the P2 mRNA (600 nucleotides: Rich & Steitz, 1987). This indicates that of the multiple loci seen in Southern blots, either only one locus is transcriptionally active, or if more than one locus is active, that only one size of transcription product is made. The results shown in Figure 3 indicate that the P2 mRNA is moderately abundant in breast tumours.

### Discussion

We have used the metastasis-associated cDNA pLM59 isolated by Elvin *et al.* (1988) to identify two homologous clones from a breast carcinoma cDNA library. Sequence analysis of these clones has shown them to be almost identical to the sequence encoding the human ribosomal phosphoprotein P2 (Rich & Steitz, 1987). The pLM59 cDNA

**Figure 2** The genomic organisation of pLM59-homologous sequences as shown by Southern analysis. This example shows (**a**) breast carcinoma DNA samples and DNA from the corresponding lymph node metastases (4 μg) digested with *Eco*RI and probed with pLM59 (see Materials and methods). The *Eco*RI RFLP is seen as the appearance of a band of 8.8 kbp in certain tumours. When this band is present in the primary tumour it is also present in the lymph node metastasis. C, carcinoma; L, lymph node metastasis. There is also a *Hin*dIII RFLP (**b**) as seen here in two examples of colonic carcinoma. The 8.0 kbp and the 4.7 kbp *Hin*dIII fragments seen with the pLM59 probe are allelic. Both RFLPs are present in the DNA from normal individuals, and neither RFLP correlates with carcinoma of the breast or colon. Sizes are in kilobase pairs.

**Figure 3** Expression of the P2 gene(s) in breast fibroadenomas and carcinomas, as shown by Northern blotting. **a**, 10 μg of total RNA was electrophoresed and blotted according to Materials and methods, and the filters were probed with pLM59. After washing in 2 × SSC at 65°C the filters were autoradiographed at −70°C for 60 h. The filters were then stripped and rehybridised to the mouse 7S RNA (Balmain *et al.*, 1982) as an internal control for the amount of RNA on the filters (**b**). F, fibroadenomas of the breast; C, breast carcinomas. The size of the P2 transcript is shown in nucleotides.

**Figure 4** Relative levels of expression of P2 gene(s) in the breast fibroadenoma and carcinoma samples shown in Figure 3. Laser scanning densitometry data were adjusted according to the level of signal with the 7S RNA probe, and expressed as a percentage of the highest signal obtained. F, fibroadenoma (hatched bars); C, carcinoma (solid bars).

probe detects a single mRNA species of 630 nucleotides, by Northern analysis (Figure 3). In the tissue samples used in this study (seven breast carcinomas, seven fibroadenomas of the breast), the abundance of pLM59-homologous mRNA in fibroadenomas was significantly higher than in carcinomas (Student's $t$ test, one tail; $P \leqslant 0.01$), with an average of 5.4-fold higher levels (Figure 4). This is in contrast to the results obtained by Elvin *et al.* (1988), who found that the expression of pLM59 is enhanced in a liver metastasis compared with a primary colon carcinoma and normal colonic mucosae.

In addition to these expression data, the cDNA library from which C328-15 and C328-16 were isolated had undergone two rounds of differential screening with probes derived from one carcinoma and one fibroadenoma. In this differential screening, the pLM59-homologous mRNA appeared to be more highly abundant in the fibroadenoma-derived probe than in the carcinoma-derived probe. These results seem paradoxical as the pLM59 cDNA was expected to be more highly expressed in the malignant tumour samples, as is the case with colonic tissue, than in the benign breast tissue. It is difficult to make comparisons between different tissue types concerning levels of gene expression. The routes along which different tissues, and indeed individual tumours in the same tissue, progress towards malignancy may vary significantly and be reflected in different profiles of gene expression. There is a degree of overlap in the abundance of pLM59-homologous mRNA in both the breast carcinomas and the fibroadenomas in this study, and in the primary colon carcinomas and secondary tumours examined by Elvin *et al.* (1988). Larger sample sizes are required to predict accurately whether pLM59 may or may not be implicated in these and other human tumours.

Southern analysis using the P2 cDNA, pLM59, revealed a complex genomic organisation suggestive of either multiple copies of the P2 gene, the presence of pseudogenes or a family of related genes (Figure 2). Which of these possibilities is the case has yet to be resolved. There are also RFLPs in *Eco*RI- and *Hin*dIII-digested DNA from a proportion of all individuals, but the presence of the RFLP does not correlate with the primary tumours of breast or colon, or the metastases from these carcinomas.

The ribosomal phosphoprotein P2 is the functional counterpart of the eL12 protein from *Artemia salina* (Amons *et al.*, 1979), the P2 protein of rat (Lin *et al.*, 1982) and the YPA1 (YL44c) protein of *Saccharomyces cerevisiae* ribosomes (Itoh, 1981). The bacterial equivalent in *Escherichia coli* is the L7/L12 protein. These phosphoproteins are collectively called 'P' or 'A' proteins, because they are highly acidic, which is unusual for ribosomal proteins. P-proteins exist as dimers in solution, and appear on the ribosome as two dimers – the only polypeptides to be present in more than single copies per ribosome (for reviews see Möller & Maassen, 1986; Liljas *et al.*, 1986). P-proteins have a distinctive amino acid composition: more than 20% alanine, only one or two arginines and generally no cysteine or tryptophan residues. The carboxy-terminal 17 amino acids of all P-proteins that have been sequenced are very highly conserved (84%) between yeast and man (Itoh, 1981; Rich & Steitz, 1987) (see Figure 1). It is this conserved region which is the antigenic determinant in some patients with the autoimmune disease systemic lupus erythematosus (Elkon *et al.*, 1986).

Of all P-proteins the L7/L12 protein from *E.coli* is the best studied. It is involved in the interaction of the ribosome with several translation factors, including the initiation factor IF2, the elongation factors EF-Tu and EF-G, and the release factors RF1 and RF2 (Weissbach, 1980). The eukaryotic counterparts P1 and P2 are similar in these respects, and also in their requirement in whole ribosomes for aminoacyl-tRNA binding and EF2-dependent GTPase activity, as well as polypeptide synthesis (MacConnell & Kaplan, 1980, 1982). There is some evidence that only the phosphorylated forms of the P-proteins can bind to the ribosome in yeast (Vidales *et al.*, 1984; Sánchez-Madrid *et al.*, 1985), and that only phosphorylated proteins can successfully reconstitute rat liver ribosomes (MacConnell & Kaplan, 1982; Lavergne *et al.*, 1987). Vidales *et al.* (1984) also allude to the effect of perturbations in metabolism being associated with changes in the proportion of phosphorylated and non-phosphorylated P-proteins in the ribosome-associated and cytoplasmic pools. If the P-proteins only function in their phosphorylated state, then it is possible that disruption of the normal regulatory pathway for the phosphorylation event could affect the peptide elongation process. If the level of phosphorylation of these proteins is one regulatory mechanism for the overall rate of protein elongation, then it is conceivable that changes in the rate of production of these proteins could upset the normal control of the protein synthesis. Comparisons can be drawn with the 40S subunit protein S6, which is phosphorylated during periods of tissue regeneration, development, cell growth and transformation (Thomas, 1986, and references therein). This protein is also phosphorylated in response to various growth factors and purified tyrosine-specific protein kinases (Thomas *et al.*, 1982; Wettenhall *et al.*, 1982; Martin-Pérez *et al.*, 1984; Maller *et al.*, 1985). Phosphorylated 40S subunits appear to be utilised more efficiently in the formation of initiation complexes (Duncan & McConkey, 1982).

Enhanced expression of ribosomal proteins could be indicative of a higher rate of overall translation, which may be related to the proliferation rate of the cells under investigation. The proliferation rate of the carcinomas used in this study has been examined previously, and was shown to be high for six out of the seven carcinomas used (Walker & Camplejohn, 1986) (Table I). Using DNA flow cytometry, tumours were classified as being highly proliferative when more than 14% of cells were in S-phase. Only tumour C3 was classified as medium by this method (Table I). Meyer (1977) has examined the proliferation rates of fibroadenomas and carcinomas of the breast by measuring the nuclear incorporation of tritiated thymidine. He found that the mitotic activity of fibroadenomas is similar to that of normal breast tissue, and lower than that of carcinomas. The proliferative rate of fibroadenomas is negatively correlated with the age of the patient (Meyer, 1977). In this study, the ages of the patients from which the fibroadenomas were taken ranged between 17 and 40 years and no correlation was found between expression of pLM59-homologous mRNA and age. The growth rate of fibroadenomas often exceeds carcinomas, but this can be explained by differences in cell longevity (Meyer, 1977). It therefore seems unlikely that the increased level of pLM59 expression seen here in fibroadenomas is due to the rates of proliferation of the tumour types compared.

**Table I** Selected properties of primary breast carcinomas used in this study

| Tumour | Grade[a] | *Node status*[b] | *Menopausal status*[c] | S-phase[d] |
|---|---|---|---|---|
| C1 | II | + | pre | 14.3 |
| C2 | III | n.k. | peri/post | 14.0 |
| C3 | III | + | post | 13.1 |
| C4 | III | + | peri/post | 21.0 |
| C5 | III | + | post | 20.0 |
| C6 | III | + | n.k. | 17.0 |
| C7 | II | + | pre | high |

[a]Tumours were graded histologically according to a modification of the Bloom and Richardson (1957) grading system (Elston *et al.*, 1982). [b]Node status: + spread to axillary nodes; n.k., not known. [c]Menopausal status defined as pre-, peri- or post-menopausal; n.k. not known. [d]S-phase levels are shown as a percentage of tumour cells in S-phase. Low 0–7%; medium 7–14%; high over 14%. Data are taken from Varley *et al.* (1987), where C1 is C298; C2 is C300; C3 is C301; C4 is C312; C5 is C317; C6 is C336; and C7 is C378.

The tumour samples used in the construction of the cDNA libraries and in the screening procedures contained a certain amount of infiltrating normal stromal cells and lymphocytes. These cells would contribute to the patterns of expression observed, and so may mask the underlying expression patterns in the tumour cells. The stromal lymphocytic infiltrate has been assayed for the carcinomas used in this study, and was found to be uniformly low (Whittaker *et al.*, 1986). In addition, the degree of differentiation, node status and menopausal status are shown in Table I. Lymphocytic infiltrate in the fibroadenomas was also very low and so the relative levels of expression of P2 described here for breast biopsies are probably not due to the stromal content of the samples. The tumours were selected primarily on the size, cellularity and therefore the amount of RNA available from the biopsy. Within this group of tumours, both grade 3 and grade 2 tumours were assayed for pLM59 expression, and no correlation with tumour grade was observed.

The possible mechanism by which altered expression of P2 could be involved with the capability of a cell to metastasise is unclear at the present. Much more work on the regulation in normal cells of both expression and function of P-proteins and the changes that accompany transformation and progression is needed in order to fully understand whether or not these proteins play a role in human cancer.

We are very grateful to Ms Angie Phanopoulous and Ms Jackie Swallow for excellent technical assistance, and also to G.D. Birnie and C.S.M. McArdle for their contributions to the early stages of this work.

### References

AMONS, R., PLUIJMS, W., KRIEK, J. & MÖLLER, W. (1982). The primary structure of protein eL12′/eL12′-*P* from the large subunit of *Artemia salina* ribosomes. *FEBS Lett.*, **146,** 143.

AMONS, R., PLUIJMS, W. & MÖLLER, W. (1979). The primary structure of ribosomal protein eL12/eL12-*P* from *Artemia salina* 80S ribosomes. *FEBS Lett.*, **104,** 85.

BALMAIN, A., KRUMLAUF, R., VASS, J.K. & BIRNIE, G.D. (1982). Cloning and characterisation of the abundant cytoplasmic 7S RNA from mouse cells. *Nucl. Acids Res.*, **10,** 4259.

BIRNBOIM, H.C. & DOLY, J. (1979). A rapid alkaline extraction procedure for screening recombinant plasmid DNA. *Nucl. Acids Res.*, **7,** 1513.

BLAMEY, R.W., DAVIES, C.J., ELSTON, C.W., JOHNSON, J., HAYBITTLE, J.L. & MAYNARD, P.V. (1979). Prognostic factors in breast cancer: The formation of a prognostic index. *Clin. Oncol.*, **5,** 227.

BLOOM, H.J.G. & RICHARDSON, W. (1957). Histological grading and prognosis in breast cancer. *Br. J. Cancer*, **11,** 359.

BRINKLEY, D. & HAYBITTLE, J.L. (1975). The curability of breast cancer. *Lancet*, **ii,** 95.

DEAR, T.N., RAMSHAW, I.A & KEFFORD, R.F. (1988). Differential expression of a novel gene, WDNM1, in nonmetastatic rat mammary adenocarcinoma cells. *Cancer Res.*, **48**, 5203.

DUNCAN, R. & MCCONKEY, E.H. (1982). Preferential utilization of phosphorylated 40-S ribosomal subunits during initiation complex formation. *Eur. J. Biochem.*, **123**, 535.

ELKON, K., SKELLY, S., PARNASSA, A. & 4 others (1986). Identification and chemical synthesis of a ribosomal protein antigenic determinant in systemic lupus erythematosus. *Proc. Natl Acad. Sci. USA*, **83**, 7419.

ELSTON, C.W., GRESHAM, G.A., RAO, G.S. & 4 others (1982). The Cancer Research Campaign (Kings/Cambridge) trial for early breast cancer: clinico-pathological aspects. *Br. J. Cancer*, **45**, 655.

ELVIN, P., KERR, I.B., MCARDLE, C.S. & BIRNIE, G.D. (1988). Isolation and preliminary characterisation of cDNA clones representing mRNAs associated with tumour progression and metastasis in colorectal cancer. *Br. J. Cancer*, **57**, 36.

FEINBERG, A.P. & VOGELSTEIN, B. (1983). A technique for radiolabeling DNA restriction endonuclease fragments to high specific activity. *Anal. Biochem.*, **132**, 6.

FIDLER, I.J. & HART, I.R. (1982). Biological diversity in metastatic neoplasms: origins and implications. *Science*, **217**, 998.

FISHER, E.R., SASS, R., FISHER, B. (1984). Pathologic findings from the National Surgical Adjuvant Project for breast cancer (protocol no. 4). Discriminants for tenth year treatment failure. *Cancer*, **53**, 712.

GRUNSTEIN, M. & HOGNESS, D.S. (1975). Colony hybridization: a method for the isolation of cloned DNAs that contain a specific gene. *Proc. Natl Acad. Sci. USA*, **72**, 3961.

GUBLER, U. & HOFFMAN, B.J. (1983). A simple and very efficient method for generating cDNA libraries. *Gene*, **25**, 263.

HEPPNER, G.H., LOVELESS, S.E., MILLER, F.R., MAHONY, K.H. & FULTON, A.M. (1984). Mammary tumor heterogeneity. In *Cancer Invasion and Metastasis: Biologic and Therapeutic Aspects*, Nicolson, G.L. & Milas, L. (eds), p. 209. Raven Press: New York.

HERMANEK, P. & SOBIN, L.H. (eds) (1987). *TNM Classification of Malignant Tumours*, 4th edition. Springer-Verlag: Berlin.

ITOH, T. (1981). Primary structure of an acidic ribosomal phosphoprotein YPA1 from *Saccharomyces cerevisiae*. *Biochim. Biophys. Acta*, **671**, 16.

LAVERGNE, J.-P., CONQUET, F., REBOUD, J.-P. & REBOUD, A.-M. (1987). Role of acidic phosphoproteins in the partial reconstitution of the active 60S ribosomal subunit. *FEBS Lett.*, **216**, 83.

LILJAS, A., KIRSEBOM, L.A. & LEIJONMARCK, M. (1986). Structural studies of the factor binding domain. In *Structure, Function, and Genetics of Ribosomes*, Hardesty, B. & Kramer, G. (eds), p. 379. Springer-Verlag: New York.

LIN, A., WITTMANN-LIEBOLD, B., MCNALLY, J. & WOOL, I.G. (1982). The primary structure of the acidic phosphoprotein P2 from rat liver 60S ribosomal subunits. *J. Biol. Chem.*, **257**, 9189.

LIOTTA, L. (1986). Tumour invasion and metastasis – role of the extracellular matrix. Rhoads Memorial Award Lecture. *Cancer Res.*, **46**, 1.

MAASSEN, J.A., SCHOP, E.N., BRANDS, J.H.G.M., VAN HEMERT, F.J., LENSTRA, J.A. & MÖLLER, W. (1985). Molecular cloning and analysis of cDNA sequences for two ribosomal proteins from *Artemia*. *Eur. J. Biochem.*, **149**, 609.

MACCONNELL, W.P. & KAPLAN, N.O. (1980). The role of ethanol extractable proteins from the 80S rat liver ribosome. *Biochim. Biophys. Acta*, **92**, 46.

MACCONNELL, W.P. & KAPLAN, N.O. (1982). The activity of the acidic phosphoproteins from the 80S rat liver ribosome. *J. Biol. Chem.*, **257**, 5359.

MALLER, J.L., FOULKES, J.G., ERIKSON, E. & BALTIMORE, D. (1985). Phosphorylation of ribosomal protein S6 on serine after microinjection of the Abelson murine leukemia virus tyrosine-specific protein kinase into *Xenopus* oocytes. *Proc. Natl Acad. Sci. USA*, **82**, 272.

MANIATIS, T., FRITSCH, E.F. & SAMBROOK, J. (1982). *Molecular Cloning – a Laboratory Manual*. Cold Spring Harbor Laboratory Press: New York.

MARTIN-PÉREZ, J., SIEGMANN, M. & THOMAS, G. (1984). EGF, $PGF_{2\alpha}$ and insulin induce the phosphorylation of identical S6 peptides in Swiss Mouse 3T3 cells: effect of cAMP on early sites of phosphorylation. *Cell*, **36**, 287.

MEYER, J.S. (1977). Cell proliferation in normal human breast ducts, fibroadenomas, and other ductal hyperplasias measured by nuclear labeling with tritiated thymidine. *Hum. Pathol.*, **8**, 67.

MÖLLER, W. & MAASSEN, J.A. (1986). On the structure, function, and dynamics of L7/L12 from *Escherichia coli* ribosomes. In *Structure, Function and Genetics of Ribosomes*, Hardesty, B. & Kramer, G. (eds), p. 309. Springer-Verlag: New York.

NOWELL, P.C. (1976). The clonal evolution of tumour cell populations. *Science*, **194**, 23.

NOWELL, P.C. (1986). Mechanisms of tumour progression. *Cancer Res.*, **46**, 2203.

RICH, B.E. & STEITZ, J.A. (1987). Human acidic ribosomal phosphoproteins P0, P1, and P2: analysis of cDNA clones, *in vitro* synthesis and assembly. *Mol. Cell. Biol.*, **7**, 4065.

SÁNCHEZ-MADRID, F., VIDALES, F.J. & BALLESTA, J.P.G. (1985). Effect of phosphorylation on the affinity of acidic proteins from *Saccharomyces cerevisiae* for the ribosomes. *Eur. J. Biochem.*, **114**, 609.

SANGER, F., NICKLEN, S. & COULSON, A.R. (1977). DNA sequencing with chain terminating inhibitors. *Proc. Natl Acad. Sci. USA*, **74**, 5463.

SCHALKEN, J.A., EBELING, S.B., ISAACS, J.T. & 4 others (1988). Down modulation of fibronectin messenger RNA in metastasizing rat prostatic cancer cells revealed by differential hybridization analysis. *Cancer Res.*, **48**, 2042.

STEEG, P.S., BEVILACQUA, G., KOPPER, L. & 4 others (1988). Evidence for a novel gene associated with low tumor metastatic potential. *J. Natl Cancer Inst.*, **80**, 200.

SHIOSAKA, T. & SAUNDERS, G.F. (1982). Differential expression of selected genes in human leukemia leukocytes. *Proc. Natl Acad. Sci. USA*, **79**, 4668.

THOMAS, G., MARTIN-PÉREZ, J., SIEGMANN, M. & OTTO, A.M. (1982). The effect of serum, EGF, $PGF_{2\alpha}$ and insulin on S6 phosphorylation and the initiation of protein and DNA synthesis. *Cell*, **30**, 235.

THOMAS, G. (1986). Epidermal growth-factor mediation of S6 phosphorylation during the mitogenic response: a novel S6 kinase. In *Oncogenes and Growth Control*, Khan, P. & Graf, T. (eds), p. 177. Springer-Verlag: Berlin.

VARLEY, J.M., SWALLOW, J.E., BRAMMAR, W.J., WHITTAKER, J.L. & WALKER, R.A. (1987). Alterations to either c-*erb*B-2 (*neu*) or c-*myc* proto-oncogenes in breast carcinomas correlate with poor short-term prognosis. *Oncogene*, **1**, 423.

VIDALES, F.J., ROBLES, M.T.S. & BALLESTA, J.P.G. (1984). Acidic proteins of the large ribosomal subunit in *Saccharomyces cerevisiae*. Effect of phosphorylation. *Biochemistry*, **23**, 390.

WALKER, R.A. & CAMPLEJOHN, R.S. (1986). DNA flow cytometry of human breast carcinomas and its relationship to transferrin and epidermal growth factor receptors. *J. Pathol.*, **150**, 37.

WEISSBACH, H. (1980). Soluble factors in protein synthesis. In *Ribosomes: Structure, Function and Genetics*, Chambliss, G., Craven, G.R., Davies, J., Davis, K., Kahan, L. & Nomura, M. (eds). University Park Press: Baltimore.

WETTENHALL, R.E.H., COHEN, P., CAUDWELL, B. & HOLLAND, R. (1982). Differential phosphorylation of ribosomal protein S6 in isolated rat hepatocytes after incubation with insulin and glucagon. *FEBS Lett.*, **148**, 207.

WHITTAKER, J.L., WALKER, R.A. & VARLEY, J.M. (1986). Differential expression of cellular oncogenes in benign and malignant human breast tissue. *Int. J. Cancer*, **38**, 651.